# A Dual Hesitant Fuzzy Multigranulation Rough Set over Two-Universe Model for Medical Diagnoses

**DOI:** 10.1155/2015/292710

**Published:** 2015-12-09

**Authors:** Chao Zhang, Deyu Li, Yan Yan

**Affiliations:** ^1^School of Computer and Information Technology, Shanxi University, Taiyuan, Shanxi 030006, China; ^2^Key Laboratory of Computational Intelligence and Chinese Information Processing of Ministry of Education, Taiyuan, Shanxi 030006, China; ^3^School and Hospital of Stomatology, Peking University, Beijing 100089, China

## Abstract

In medical science, disease diagnosis is one of the difficult tasks for medical experts who are confronted with challenges in dealing with a lot of uncertain medical information. And different medical experts might express their own thought about the medical knowledge base which slightly differs from other medical experts. Thus, to solve the problems of uncertain data analysis and group decision making in disease diagnoses, we propose a new rough set model called dual hesitant fuzzy multigranulation rough set over two universes by combining the dual hesitant fuzzy set and multigranulation rough set theories. In the framework of our study, both the definition and some basic properties of the proposed model are presented. Finally, we give a general approach which is applied to a decision making problem in disease diagnoses, and the effectiveness of the approach is demonstrated by a numerical example.

## 1. Introduction

In real-life disease diagnoses, due to the inherent uncertainty of human's expression of preferences, and the management, storage, and extraction of various useful information available to physicians which is not always presented as crisp numbers, it is believed that fuzzy numbers own many advantages for dealing with medical information systems. Moreover, in order to seek a diagnosis for the considered patients, it is essential for physicians to take into account a number of symptoms at the same time; this process might take a long time to reach a final conclusion. What is worse, the situation of overlooking a few trivial symptoms may trigger wrong disease diagnosis. To solve this complex decision making problem, lots of efforts have been made based on combining the uncertain decision making methods with the traditional disease diagnosis study. Fuzzy set theory [1], proposed by Zadeh in 1965, provides robust solutions in many application domains. In the concept of fuzzy sets, the membership degree of an element is a single crisp value within [0,1]. However, to cope with imperfect and uncertain information induced by several sources of vagueness, the classical fuzzy set is confronted with some limitations. Thus, many extension forms of fuzzy sets have been introduced and utilized in disease diagnoses [2–5].

Among the numerous decision making processes, we often encounter such situations in which decision makers hesitate among several possible membership values when determining the membership of an element belonging to a given set. To address this issue, Torra [6] and Torra and Narukawa [7] introduced the concept of hesitant fuzzy set (HFS) which has been proved useful to deal with uncertain information in multiattribute decision making procedures. We could illustrate the above-mentioned motivation in the following example. Suppose there is an expert who intends to determine the membership degree of whether a house is beautiful. The expert may consider that the membership degree is 0.7, while he holds a view that 0.8 is also justifiable. The hesitant fuzzy set is useful in the above case when the membership degree of *x* can be expressed as {0.7,0.8}. Ever since the establishment of the hesitant fuzzy set theory, many researchers have studied the HFS from various facets and obtained an increasing number of achievements. In the extensions of hesitant fuzzy set, Zhu et al. introduced the concept of dual hesitant fuzzy set (DHFS) in 2012 [8], which includes the fuzzy set, intuitionistic fuzzy set, hesitant fuzzy set, and multifuzzy set as special cases. The DHFS is described by the membership hesitancy and nonmembership hesitancy functions. It is evident that the DHFS can reflect the human's hesitancy more objectively than the other widely developed fuzzy set approaches. Thereafter, many scholars have studied the DHFS from different angles and obtained plenty of meaningful results [9–13].

In addition, rough set theory, proposed by Pawlak [14], is a well-established mechanism for dealing with uncertainty in data analysis. The rough set theory has been widely employed in many domains such as medical diagnosis, formal concept analysis, feature selection, and uncertainty reasoning [15–18]. The basic structure of rough set is an approximation space consisting of a universe of discourse and a binary relation imposed on it. In classical approximation space, the equivalence relation is a very restrictive condition, so the application domain of rough set model is narrowed, to some extent. Thus, various extension forms of classical rough set have been introduced over the past years. In the extension of universe, since the rough set on single universe may limit the description of decision information provided by experts, two or multiple universes can describe the real-world information more effectively and reasonably. Thus, the model of rough set over two universes has been studied extensively and applied in many real-life decision making problems [19–23]. For example, Pei and Xu [19] studied rough set over two-universe model and researched its properties in detail. Sun and Ma [20] studied fuzzy rough set over two universes and its related applications. Yang et al. [21] proposed a fuzzy probabilistic rough set model over two universes and utilized the model in a clinical diagnosis case. Luo and Xu [22] introduced a rough Atanassov's intuitionistic fuzzy set model over two different universes and discussed a problem about how to arrange patients to see the doctor reasonably. Sun et al. [23] utilized the model of fuzzy rough set theory over two universes under the background of the emergency material demand predictions.

Considering the view of granular computing [24], the set approximations in the above rough set theory are described by a single binary relation on a given universe. However, it is beneficial to view a problem through multiple binary relations in multigranulation backgrounds. To address the situation, Qian et al. proposed the model of multigranulation rough set (MGRS) by taking multiple binary relations into account [25, 26]. And two types of multigranulation rough set, that is, optimistic multigranulation rough set based on “seeking common ground while reserving differences” (SCRD) strategy and pessimistic multigranulation rough set based on “seeking common ground while eliminating differences” (SCED) strategy, were introduced [27]. Moreover, several extension forms of multigranulation rough set have been put forward during these years [28–33]. Among them, Sun and Ma [33] proposed the multigranulation rough set over two universes and discussed its properties and some uncertainty measures. The multigranulation rough set over two universes owns some superiorities in group decision making. In information fusion procedures, in order to enhance the decision level, we usually need to obtain the optimal solutions according to the assessment information provided by multiple experts. And different expert often views the decision making problems from different angles, but they share a common goal in reaching a final agreement that synthesizes each expert's opinion. Thus, the method of multigranulation rough set over two universes is an ideal information fusion strategy which could synthesize each decision maker's view to form a final decision.

In this paper, we propose the dual hesitant fuzzy (DHF) multigranulation rough set over two-universe model by combining the dual hesitant fuzzy set and multigranulation rough set over two universes. Both the general definition and some useful properties of the proposed model will be discussed. Then, we explore a new approach to the decision making problem in medical diagnoses by utilizing the DHF multigranulation rough set over two-universe model. Finally, we give an illustrative example to verify the developed approach and demonstrate its validity and feasibility.

The remaining part of this paper is organized as follows. In Section 2, we present the basic knowledge about hesitant fuzzy sets, dual hesitant fuzzy sets, rough set over two universes, and multigranulation rough set over two universes. In Section 3, we introduce the DHF multigranulation rough set over two universes and some properties are discussed.  Section 4 presents an approach to the decision making problem in medical diagnoses by utilizing the proposed model. In Section 5, we illustrate the steps of the proposed decision making method by a numerical example. In Section 6, we conclude this paper with some remarks.

## 2. Preliminaries

In this section, we first review some basic concepts such as hesitant fuzzy sets, dual hesitant fuzzy sets, and their properties. Then we present the definition of rough set over two universes and multigranulation rough set over two universes.

### 2.1. Hesitant Fuzzy Sets

Hesitant fuzzy sets (HFSs) were introduced by Torra [6] and Torra and Narukawa [7], which permit the membership degree of an element to a reference set expressed by several possible values between 0 and 1.

**Definition 1 (see [7]).**

Let *U* be the universe of discourse; a hesitant fuzzy set *F* on *U* is defined as a function *h*_*F*_(*x*) that returns a subset of [0,1], which can be expressed as the following mathematical symbol:

(1) $$\begin{array}{c} F=\left\{\;\langle \;x,h_{F}\left(\;x\;\right)\;\rangle \;|\;x\in U\;\right\}, \end{array}$$

where *h*_*F*_(*x*) is a set of some different finite values in [0,1], describing the possible membership degrees of the element *x* ∈ *U* to the set *F*. For convenience, *h*_*F*_(*x*) is called a hesitant fuzzy element. The set of all hesitant fuzzy elements is called HFEs.

### 2.2. Dual Hesitant Fuzzy Sets

Zhu et al. [8] further extended the concept of HFSs to develop the dual hesitant fuzzy sets (DHFSs), which are defined in terms of two functions which returns two sets of membership values and nonmembership values, respectively.

**Definition 2 (see [8]).**

Let *U* be the universe of discourse; then a dual hesitant fuzzy set *D* on *U* is defined as

(2) $$\begin{array}{c} D=\left\{\;\langle \;x,h\left(\;x\;\right),g\left(\;x\;\right)\;\rangle \;|\;x\in U\;\right\}, \end{array}$$

where *h*(*x*) and *g*(*x*) are two sets of some different finite values in [0,1], describing the possible membership degrees and nonmembership degrees of the element *x* ∈ *U* to the set *D*, respectively, with the conditions 0 ≤ *γ*, *η* ≤ 1, 0 ≤ *γ* ^+^ + *η* ^+^ ≤ 1, where *γ* ∈ *h*(*x*), *η* ∈ *g*(*x*), *γ* ^+^ ∈ *h* ^+^(*x*) = ∪_*γ*∈*h*(*x*)_max⁡{*γ*}, and *η* ^+^ ∈ *g* ^+^(*x*) = ∪_*η*∈*g*(*x*)_max⁡{*η*} for all *x* ∈ *U*. For convenience, the pair *d*(*x*) = (*h*(*x*), *g*(*x*)) is called a dual hesitant fuzzy element. The set of all dual hesitant fuzzy elements is called DHFEs.

Suppose that *U* is the universe of discourse; then the set of all dual hesitant fuzzy sets on *U* is denoted by DHF(*U*).

**Example 3 .**

Let *U* = {*x*_1_, *x*_2_} be a universe set; then a dual hesitant fuzzy set defined by *D* = {〈*x*_1_, {0.6,0.7}, {0.2,0.3}〉, 〈*x*_2_, {0.3,0.4}, {0.5,0.6}〉} is a dual hesitant fuzzy set.

Here, we present two special dual hesitant fuzzy sets as follows:

(1) *D*  is referred to as an empty dual hesitant fuzzy set [8] if and only if *h*(*x*) = {0} and *g*(*x*) = {1} for all *x* ∈ *U*. In that case, the empty dual hesitant fuzzy set is denoted by *∅* in this paper.
(2) *D* is referred to as a full dual hesitant fuzzy set [8] if and only if *h*(*x*) = {1} and *g*(*x*) = {0} for all *x* ∈ *U*. In that case, the full dual hesitant fuzzy set is denoted by *U* in this paper.

Similar to hesitant fuzzy set theory, Zhu et al. [8] also defined the complement, union, and intersection operations on dual hesitant fuzzy set as follows.

**Definition 4 (see [8]).**

Let *U* be the universe of discourse, ∀*D*, *D*_1_, *D*_2_ ∈ DHF(*U*). Then, the complement, union, and intersection operations are defined as follows:

(1) The complement of *D*, denoted by *D* ^*c*^, is defined as
  (3) dx=∪γ∈hx,η∈gxη,γ,if  gx≠∅,  hx≠∅∪γ∈hx1−γ,∅,if  gx=∅,  hx≠∅∪η∈gx∅,1−η,if  gx≠∅,  hx=∅.
(2) The union of *D*_1_ and *D*_2_, denoted by *D*_1_ ∪ *D*_2_, is defined as *d*_1_(*x*)∨*d*_2_(*x*) = {*h*(*x*) ∈ (*h*_1_(*x*) ∪ *h*_2_(*x*))∣*h*(*x*)≥ max⁡(*h*_1_^−^(*x*), *h*_2_^−^(*x*)), *g*(*x*) ∈ (*g*_1_(*x*) ∪ *g*_2_(*x*))∣*g*(*x*) ≤ min⁡(*g*_1_^+^(*x*), *g*_2_^+^(*x*))}.
(3) The intersection of *D*_1_ and *D*_2_, denoted by *D*_1_∩*D*_2_, is defined as *d*_1_(*x*)∧*d*_2_(*x*) = {*h*(*x*) ∈ (*h*_1_(*x*) ∪ *h*_2_(*x*))∣*h*(*x*)≤ min⁡(*h*_1_^+^(*x*), *h*_2_^+^(*x*)), *g*(*x*) ∈ (*g*_1_(*x*) ∪ *g*_2_(*x*))∣*g*(*x*)≥ max⁡(*g*_1_^−^(*x*), *g*_2_^−^(*x*))},

where *d*_1_(*x*) and *d*_2_(*x*) are DHFEs of two DHFSs, *D*_1_ and *D*_2_, respectively. Moreover, *h* ^−^(*x*), *h* ^+^(*x*), *g* ^−^(*x*), and *g* ^+^(*x*) are lower and upper bounds of *h*(*x*) and *g*(*x*), respectively. Among them, *h* ^−^(*x*) = ∪_*γ*∈*h*(*x*)_min⁡{*γ*}, *h* ^+^(*x*) = ∪_*γ*∈*h*(*x*)_max⁡{*γ*}, *g* ^−^(*x*) = ∪_*η*∈*g*(*x*)_min⁡{*η*}, and *g* ^+^(*x*) = ∪_*η*∈*g*(*x*)_max⁡{*η*}.

It should be noted in above definition that the operations ^*c*^, ∪, and ∩ are defined on dual hesitant fuzzy sets, respectively, while the operations ~, ∨, and ∧ are defined on corresponding dual hesitant fuzzy elements, respectively. In what follows, we present the properties of the above operations as follows.

**Theorem 5 .**

Let *U* be the universe of discourse, ∀*D*, *D*_1_, *D*_2_, *D*_3_ ∈ *DHF*(*U*). Then the following properties are true:

(1) Double negation law: (*D* ^*c*^)^*c*^ = *D*.
(2) De Morgan's laws: (*D*_1_ ∪ *D*_2_)^*c*^ = *D*_1_^*c*^∩*D*_2_^*c*^, (*D*_1_∩*D*_2_)^*c*^ = *D*_1_^*c*^ ∪ *D*_2_^*c*^.
(3) Commutativity: *D*_1_ ∪ *D*_2_ = *D*_2_ ∪ *D*_1_, *D*_1_∩*D*_2_ = *D*_2_∩*D*_1_.
(4) Associativity: *D*_1_ ∪ (*D*_2_ ∪ *D*_3_) = (*D*_1_ ∪ *D*_2_) ∪ *D*_3_, *D*_1_∩(*D*_2_∩*D*_3_) =  (*D*_1_∩*D*_2_)∩*D*_3_.
(5) Distributivity: *D*_1_ ∪ (*D*_2_∩*D*_3_) = (*D*_1_ ∪ *D*_2_)∩(*D*_1_ ∪ *D*_3_), *D*_1_∩(*D*_2_ ∪ *D*_3_) = (*D*_1_∩*D*_2_) ∪ (*D*_1_∩*D*_3_).

**Proof**

It can be obtained directly from Definition 4.

**Example 6 .**

Let *d*_1_(*x*) = {{0.1,0.4,0.5}, {0.2,0.3}} and *d*_2_(*x*) = {{0.2,0.3,0.4}, {0.4,0.5}} be two DHFEs; then we can obtain the complement, union, and intersection as follows:

(1) ~*d*_1_(*x*) = {{0.2,0.3}, {0.1,0.4,0.5}} and ~*d*_2_(*x*) = {{0.4,0.5}, {0.2,0.3,0.4}}.
(2) *d*_1_(*x*)∨*d*_2_(*x*) = {{0.2,0.3,0.4,0.5}, {0.2,0.3}}.
(3) *d*_1_(*x*)∧*d*_2_(*x*) = {{0.1,0.2,0.3,0.4}, {0.4,0.5}}.

To compare the magnitude of different dual hesitant fuzzy elements, Zhu et al. [8] introduced the following comparison laws.

**Definition 7 (see [8]).**

Let *d*_*i*_(*x*) = {*h*_*d*_*i*__(*x*), *g*_*d*_*i*__(*x*)}  (*i* = 1,2) be any two DHFEs. The score function of *d*_*i*_(*x*) is *S*_*d*_*i*_(*x*)_ = (1/#*h*(*x*))∑_*γ*∈*h*(*x*)_ *γ* − (1/#*g*(*x*))∑_*η*∈*g*(*x*)_ *η*, where #*h*(*x*) and #*g*(*x*) are the numbers of the elements in *h*(*x*) and *g*(*x*), respectively. Then, if *S*_*d*_1_(*x*)_ > *S*_*d*_2_(*x*)_, *d*_1_(*x*)≻*d*_2_(*x*); if *S*_*d*_1_(*x*)_ < *S*_*d*_2_(*x*)_, *d*_1_(*x*)≺*d*_2_(*x*); if *S*_*d*_1_(*x*)_ = *S*_*d*_2_(*x*)_, *d*_1_(*x*) ~ *d*_2_(*x*).

**Example 8 .**

Let *d*_1_(*x*) = {{0.7,0.8}, {0.2,0.3}} and *d*_2_(*x*) = {{0.4,0.6}, {0.2,0.3}} be two DHFEs; then we can obtain that *S*_*d*_1_(*x*)_ > *S*_*d*_2_(*x*)_; thus *d*_1_(*x*)≻*d*_2_(*x*).

In granular computing, it is noted that the hierarchy acts as a significant part. In classical set, the hierarchy is characterized by set containment. However, in the background of fuzzy set, the hierarchy is characterized by the comparisons of membership degrees. Since dual hesitant fuzzy set is a further extension form of fuzzy set, it is necessary to develop new definition for comparing two dual hesitant fuzzy sets. Different from the score function introduced in Definition 7 which aims to compare some different DHFEs, we will introduce the concept of DHF subset to compare two dual hesitant fuzzy sets.

**Definition 9 .**

Let *U* be the universe of discourse;  ∀*D*_1_, *D*_2_ ∈ DHF(*U*), *d*_1_(*x*) = (*h*_1_(*x*), *g*_1_(*x*)) and *d*_2_(*x*) = (*h*_2_(*x*), *g*_2_(*x*)) are DHFEs of *D*_1_ and *D*_2_, respectively. If *d*_1_(*x*)⪯*d*_2_(*x*) holds for each *x* ∈ *U* such that *h*_1_(*x*)⪯*h*_2_(*x*)⇔*h*_1_^*σ*(*k*)^(*x*) ≤ *h*_2_^*σ*(*k*)^(*x*) and *g*_1_(*x*)⪰*g*_2_(*x*)⇔*g*_1_^*σ*(*k*)^(*x*) ≥ *g*_2_^*σ*(*k*)^(*x*), where *h*_1_^*σ*(*k*)^(*x*) and *g*_1_^*σ*(*k*)^(*x*) denote the *k*th largest values in *h*_1_(*x*) and *g*_1_(*x*), respectively, while *h*_2_^*σ*(*k*)^(*x*) and *g*_2_^*σ*(*k*)^(*x*) denote the *k*th largest values in *h*_2_(*x*) and *g*_2_(*x*), respectively, then, *D*_1_ is referred to as a dual hesitant fuzzy subset of *D*_2_, which is denoted by *D*_1_⊆*D*_2_.

It is noted that the comparison of two DHFSs is based on the partial orders between corresponding DHFEs for all objects in the universe. And the partial orders between DHFEs are further based on the comparisons of each value in corresponding DHFEs. Thus, we can see that the comparison of two DHFSs is based on the comparisons of each value in corresponding DHFEs for all objects in the universe.

### 2.3. Rough Set over Two Universes

**Definition 10 (see [19]).**

Let *U* and *V* be two universes and let *R* be a compatibility relation from *U* to *V*. The mapping *F* : *U* ↦ 2^*V*^, for any *u* ∈ *U*, *v* ∈ *V*, and *u* → {*v* ∈ *V*∣(*u*, *v*) ∈ *R*}, is called a mapping induced by *R*. The ordered triple (*U*, *V*, *R*) is called an approximation space. The lower and upper approximations of *X*⊆*V* are defined as follows:

(4) apr_X=x∈U ∣ Fx⊆X,apr¯X=x∈U ∣ Fx∩X≠∅.

Then the pair $\left(\underline{\text{apr}}\left(X\right),\bar{\text{apr}}\left(X\right)\right)$ is called rough set over two universes of *X* with respect to (*U*, *V*, *R*).

### 2.4. Multigranulation Rough Set over Two Universes

Based on the rough set over two universes [19] and the model of multigranulation rough set proposed by Qian et al. [25], Sun and Ma [33] introduced the model of multigranulation rough set over two universes recently. In order to present the concept of multigranulation rough set over two universes, we give the definition of multigranulation approximation space over two universes at first.

**Definition 11 (see [19]).**

Let *U*, *V* be two nonempty and finite universes of discourse. *R* is a family binary compatibility relation from *U* to *V* induced by binary mapping family *F*_*i*_ : *U* → 2^*V*^, *u* ↦ {*v* ∈ *V*∣(*u*, *v*) ∈ *R*_*i*_}, and *R*_*i*_ ∈ *R*, *i* = 1,2,…, *m*. The ordered triple set (*U*, *V*, *R*) is the multigranulation approximation space over two universes.

In what follows, we introduce the definition of optimistic and pessimistic multigranulation rough sets over two universes.

**Definition 12 (see [33]).**

Let (*U*, *V*, *R*) be a multigranulation approximation space over two universes. Let *F* and *G* be two binary mappings from universes *U* to *V*. For any *X*⊆*V* the optimistic lower and upper multigranulation approximations with respect to (*U*, *V*, *R*) are defined as follows:

(5) apr_F+GOX=x∈U ∣ Fx⊆X∨Gx⊆X,apr¯F+GOX=apr_F+GOXcc.

The pair $\left({\underline{apr}}_{F+G}^{O}\left(X\right),{\bar{apr}}_{F+G}^{O}\left(X\right)\right)$ is the optimistic multigranulation rough set over two universes if ${\underline{apr}}_{F+G}^{O}\left(X\right)\neq {\bar{apr}}_{F+G}^{O}\left(X\right)$; otherwise, *X* is definable on (*U*, *V*, *R*) with respect to *F* and *G*. Moreover, the boundary region of *X* on (*U*, *V*, *R*) is defined as follows:

(6) $$\begin{array}{c} {\text{Bnd}}_{F+G}^{O}\left(\;X\;\right)={\bar{\text{apr}}}_{F+G}^{O}\left(\;X\;\right)-{\underline{\text{apr}}}_{F+G}^{O}\left(\;X\;\right). \end{array}$$

Similarly, the pessimistic lower and upper multigranulation approximations with respect to (*U*, *V*, *R*) are defined as follows:

(7) apr_F+GPX=x∈U ∣ Fx⊆X∧Gx⊆X,apr¯F+GPX=apr_F+GPXcc.

The pair $\left({\underline{apr}}_{F+G}^{P}\left(X\right),{\bar{apr}}_{F+G}^{P}\left(X\right)\right)$ is the pessimistic multigranulation rough set over two universes if ${\underline{apr}}_{F+G}^{P}\left(X\right)\neq {\bar{apr}}_{F+G}^{P}\left(X\right)$; otherwise, *X* is definable on (*U*, *V*, *R*) with respect to *F* and *G*. Moreover, the boundary region of *X* on (*U*, *V*, *R*) is defined as follows:

(8) $$\begin{array}{c} {\text{Bnd}}_{F+G}^{P}\left(\;X\;\right)={\bar{\text{apr}}}_{F+G}^{P}\left(\;X\;\right)-{\underline{\text{apr}}}_{F+G}^{P}\left(\;X\;\right). \end{array}$$

In above definition, the word “optimistic” means that, in multiple independent granular structures, at least one granular structure must satisfy the inclusion condition between an equivalence class and a target concept, while the word “pessimistic” means all granular structures must satisfy the inclusion condition between an equivalence class and a target concept.

## 3. DHF Multigranulation Rough Set over Two Universes

In this section, we discuss the concept of dual hesitant fuzzy multigranulation rough set over two universes systematically. At first, it is necessary to develop the definition of dual hesitant fuzzy rough set over two universes.

### 3.1. DHF Rough Set over Two Universes

**Definition 13 .**

Let *U*, *V* be two nonempty and finite universes of discourse. A dual hesitant fuzzy relation *R* from *U* to *V* is defined as follows:

(9) $$\begin{array}{c} R=\left\{\;\langle \;\left(\;x,y\;\right),h_{R}\left(\;x,y\;\right),g_{R}\left(\;x,y\;\right)\;\rangle \;|\;\left(\;x,y\;\right)\in U\times V\;\right\}, \end{array}$$

where *h*_*R*_(*x*, *y*) and *g*_*R*_(*x*, *y*) are two sets of some different finite values in [0,1], denoting the possible membership degrees and nonmembership degrees for all (*x*, *y*) ∈ *U* × *V*, respectively. With the conditions 0 ≤ *γ*, *η* ≤ 1, 0 ≤ *γ* ^+^ + *η* ^+^ ≤ 1, where *γ* ∈ *h*_*R*_(*x*, *y*), *η* ∈ *g*_*R*_(*x*, *y*), *γ* ^+^ ∈ *h*_*R*_^+^(*x*, *y*) = max_*γ*∈*h*_*R*_(*x*, *y*)_{*γ*}, and *η* ^+^ ∈ *g*_*R*_^+^(*x*, *y*) = max_*η*∈*g*_*R*_(*x*, *y*)_{*η*} for all (*x*, *y*) ∈ *U* × *V*. Moreover, the family of all dual hesitant fuzzy relations over *U* × *V* is denoted by DHFR(*U* × *V*).

**Definition 14 .**

Let *U*, *V* be two nonempty and finite universes of discourse and *R* ∈ DHFR(*U* × *V*); the pair (*U*, *V*, *R*) is called a dual hesitant fuzzy approximation space over two universes. For any *A* ∈ DHF(*V*), the lower and upper approximations of *A* with respect to (*U*, *V*, *R*), denoted by $\underline{R}\left(A\right)$ and $\bar{R}\left(A\right)$, are two dual hesitant fuzzy sets and are, respectively, defined as follows:

(10) R¯A=x,hR¯Ax,gR¯Ax ∣ x∈U,R_A=x,hR_Ax,gR_Ax ∣ x∈U,

where $h_{\bar{R}\left(A\right)}\left(x\right)={⋁}_{y\in V}\left\{h_{R}\left(x,y\right)\wedge h_{A}\left(y\right)\right\}$, $g_{\bar{R}\left(A\right)}\left(x\right)={⋀}_{y\in V}\left\{g_{R}\left(x,y\right)\vee g_{A}\left(y\right)\right\}$, $h_{\underline{R}\left(A\right)}\left(x\right)={⋀}_{y\in V}\left\{g_{R}\left(x,y\right)\vee h_{A}\left(y\right)\right\}$, and $g_{\underline{R}\left(A\right)}\left(x\right)={⋁}_{y\in V}\left\{h_{R}\left(x,y\right)\wedge g_{A}\left(y\right)\right\}$.

$\underline{R}\left(A\right)$ and $\bar{R}\left(A\right)$ are called the lower and upper approximations of *A* with respect to (*U*, *V*, *R*), respectively. The pair $\left(\underline{R}\left(A\right),\bar{R}\left(A\right)\right)$ is called the dual hesitant fuzzy rough set over two universes of *A* with respect to (*U*, *V*, *R*) and $\underline{R}$, $\bar{R}$ are referred to as lower and upper dual hesitant fuzzy rough approximation operators over two universes, respectively.

In what follows, based on the constructive approach to dual hesitant fuzzy rough set over two universes, we extend the dual hesitant fuzzy relation into the background of multigranulation rough set. Both the definitions and some basic properties of optimistic and pessimistic DHF multigranulation rough sets over two universes will be elaborated on.

### 3.2. Optimistic DHF Multigranulation Rough Set over Two Universes

**Definition 15 .**

Let *U*, *V* be two nonempty and finite universes of discourse and *R*_*i*_ ∈ DHFR(*U* × *V*)  (*i* = 1,2,…, *m*) are *m* dual hesitant fuzzy relations over *U* × *V*; the pair (*U*, *V*, *R*_*i*_) is called a dual hesitant fuzzy multigranulation approximation space over two universes. For any *A* ∈ DHF(*V*), the optimistic lower and upper approximations of *A* with respect to (*U*, *V*, *R*_*i*_) are defined as follows:

(11) ∑i=1mRi_OA=x,h∑i=1mRi_OAx,g∑i=1mRi_OAx ∣ x∈U,∑i=1mRi¯OA=x,h∑i=1mRi¯OAx,g∑i=1mRi¯OAx ∣ x∈U,

where $h_{{\underline{\sum_{i=1}^{m}R_{i}}}^{O}\left(A\right)}\left(x\right)={⋁}_{i=1}^{m}{⋀}_{y\in V}^{}\left\{g_{R_{i}}\left(x,y\right)\vee h_{A}\left(y\right)\right\}$, $g_{{\underline{\sum_{i=1}^{m}R_{i}}}^{O}\left(A\right)}\left(x\right)=$ ⋀_*i*=1_^*m*^⋁_*y*∈*V*_{*h*_*R*_*i*__(*x*, *y*)∧*g*_*A*_(*y*)}, $h_{{\bar{\sum_{i=1}^{m}R_{i}}}^{O}\left(A\right)}\left(x\right)=$ ⋀_*i*=1_^*m*^⋁_*y*∈*V*_{*h*_*R*_*i*__(*x*, *y*)∧*h*_*A*_(*y*)}, and $g_{{\bar{\sum_{i=1}^{m}R_{i}}}^{O}\left(A\right)}\left(x\right)=$ ⋁_*i*=1_^*m*^⋀_*y*∈*V*_{*g*_*R*_*i*__(*x*, *y*)∨*g*_*A*_(*y*)}.

We call the pair $\left({\underline{\sum_{i=1}^{m}R_{i}}}^{O}\left(A\right),{\bar{\sum_{i=1}^{m}R_{i}}}^{O}\left(A\right)\right)$ an optimistic DHF multigranulation rough set over two universes of *A* with respect to (*U*, *V*, *R*_*i*_). If ${\underline{\sum_{i=1}^{m}R_{i}}}^{O}\left(A\right)={\bar{\sum_{i=1}^{m}R_{i}}}^{O}\left(A\right)$, we call *A* optimistic-definable in (*U*, *V*, *R*_*i*_); otherwise, *A* is optimistic-undefinable in (*U*, *V*, *R*_*i*_). It is noted that the optimistic DHF multigranulation rough set over two universes will reduce to a DHF rough set over two universes if *m* = 1.

**Theorem 16 .**

Let *U*, *V* be two nonempty and finite universes of discourse and *R*_*i*_ ∈ *DHFR*(*U* × *V*)  (*i* = 1,2,…, *m*) are m dual hesitant fuzzy relations over *U* × *V*. For any *A*, *A*′ ∈ *DHF*(*V*), the optimistic DHF multigranulation rough set over two universes has the following properties:

(1) ${\underline{\sum_{i=1}^{m}R_{i}}}^{O}\left(A^{c}\right)={\left({\bar{\sum_{i=1}^{m}R_{i}}}^{O}\left(A\right)\right)}^{c}$; ${\bar{\sum_{i=1}^{m}R_{i}}}^{O}\left(A^{c}\right)={\left({\underline{\sum_{i=1}^{m}R_{i}}}^{O}\left(A\right)\right)}^{c}$.
(2) $A\subseteq A^{\prime }\Rightarrow {\underline{\sum_{i=1}^{m}R_{i}}}^{O}\left(A\right)\subseteq {\underline{\sum_{i=1}^{m}R_{i}}}^{O}\left(A^{\prime }\right)$; $A\subseteq A^{\prime }\Rightarrow {\bar{\sum_{i=1}^{m}R_{i}}}^{O}\left(A\right)\subseteq {\bar{\sum_{i=1}^{m}R_{i}}}^{O}\left(A^{\prime }\right)$.
(3) ${\underline{\sum_{i=1}^{m}R_{i}}}^{O}\left(A\cap A^{\prime }\right)={\underline{\sum_{i=1}^{m}R_{i}}}^{O}\left(A\right)\cap {\underline{\sum_{i=1}^{m}R_{i}}}^{O}\left(A^{\prime }\right)$; ${\bar{\sum_{i=1}^{m}R_{i}}}^{O}\left(A\cup A^{\prime }\right)=$ ${\bar{\sum_{i=1}^{m}R_{i}}}^{O}\left(A\right)\cup$ ${\bar{\sum_{i=1}^{m}R_{i}}}^{O}\left(A^{\prime }\right)$.
(4) ${\underline{\sum_{i=1}^{m}R_{i}}}^{O}\left(A\cup A^{\prime }\right)⊇{\underline{\sum_{i=1}^{m}R_{i}}}^{O}\left(A\right)\cup {\underline{\sum_{i=1}^{m}R_{i}}}^{O}\left(A^{\prime }\right)$; ${\bar{\sum_{i=1}^{m}R_{i}}}^{O}\left(A\cap A^{\prime }\right)\subseteq$ ${\bar{\sum_{i=1}^{m}R_{i}}}^{O}\left(A\right)\cap$ ${\bar{\sum_{i=1}^{m}R_{i}}}^{O}\left(A^{\prime }\right)$.

**Proof**

(1) For all *x* ∈ *U*, we have ${\underline{\sum_{i=1}^{m}R_{i}}}^{O}\left(A^{c}\right)=$ {〈*x*, ⋁_*i*=1_^*m*^⋀_*y*∈*V*_{*g*_*R*_*i*__(*x*, *y*)∨*h*_~*A*_(*y*)}, ⋀_*i*=1_^*m*^⋁_*y*∈*V*_{*h*_*R*_*i*__(*x*, *y*)∧*g*_~*A*_(*y*)}〉∣*x* ∈ *U*} =  {〈*x*, ⋁_*i*=1_^*m*^⋀_*y*∈*V*_{*g*_*R*_*i*__(*x*, *y*)∨*g*_*A*_(*y*)}, ⋀_*i*=1_^*m*^⋁_*y*∈*V*_{*h*_*R*_*i*__(*x*, *y*)∧*h*_*A*_(*y*)}〉∣*x* ∈ *U*} =  ${\left({\bar{\sum_{i=1}^{m}R_{i}}}^{O}\left(A\right)\right)}^{c}$. Similarly, it is not difficult to prove that ${\bar{\sum_{i=1}^{m}R_{i}}}^{O}\left(A^{c}\right)={\left({\underline{\sum_{i=1}^{m}R_{i}}}^{O}\left(A\right)\right)}^{c}$.

(2) Since *A*⊆*A*′, then by Definition 9, we have *h*_*A*_^*σ*(*k*)^(*y*) ≤ *h*_*A*′_^*σ*(*k*)^(*y*) and *g*_*A*_^*σ*(*k*)^(*y*) ≥ *g*_*A*′_^*σ*(*k*)^(*y*) for all *y* ∈ *V*. So it follows that {〈*x*, ⋁_*i*=1_^*m*^⋀_*y*∈*V*_{*g*_*R*_*i*__^*σ*(*k*)^(*x*, *y*)∨*h*_*A*_^*σ*(*k*)^(*y*)}, ⋀_*i*=1_^*m*^⋁_*y*∈*V*_{*h*_*R*_*i*__^*σ*(*k*)^(*x*, *y*)∧*g*_*A*_^*σ*(*k*)^(*y*)}〉∣*x* ∈ *U*}≤ {〈*x*, ⋁_*i*=1_^*m*^⋀_*y*∈*V*_{*g*_*R*_*i*__^*σ*(*k*)^(*x*, *y*)∨*h*_*A*′_^*σ*(*k*)^(*y*)}, ⋀_*i*=1_^*m*^⋁_*y*∈*V*_{*h*_*R*_*i*__^*σ*(*k*)^(*x*, *y*)∧*g*_*A*′_^*σ*(*k*)^(*y*)}〉∣*x* ∈ *U*}. Hence, for each *x* ∈ *U*, we have $h_{{\underline{\sum_{i=1}^{m}R_{i}}}^{O}\left(A\right)}\left(x\right)⪯h_{{\underline{\sum_{i=1}^{m}R_{i}}}^{O}\left(A^{\prime }\right)}\left(x\right)$ and $g_{{\underline{\sum_{i=1}^{m}R_{i}}}^{O}\left(A\right)}\left(x\right)⪰g_{{\underline{\sum_{i=1}^{m}R_{i}}}^{O}\left(A^{\prime }\right)}\left(x\right)$, which means ${\underline{\sum_{i=1}^{m}R_{i}}}^{O}\left(A\right)\subseteq {\underline{\sum_{i=1}^{m}R_{i}}}^{O}\left(A^{\prime }\right)$. Similarly, it is not difficult to prove that $A\subseteq A^{\prime }\Rightarrow {\bar{\sum_{i=1}^{m}R_{i}}}^{O}\left(A\right)\subseteq {\bar{\sum_{i=1}^{m}R_{i}}}^{O}\left(A^{\prime }\right)$.

(3) Consider ${\underline{\sum_{i=1}^{m}R_{i}}}^{O}\left(A\cap A^{\prime }\right)=$ {〈*x*, ⋁_*i*=1_^*m*^⋀_*y*∈*V*_{*g*_*R*_*i*__(*x*, *y*)∨*h*_*A*∩*A*′_(*y*)}, ⋀_*i*=1_^*m*^⋁_*y*∈*V*_{*h*_*R*_*i*__(*x*, *y*)∧*g*_*A*∩*A*′_(*y*)}〉∣*x* ∈ *U*} =  {〈*x*, ⋁_*i*=1_^*m*^⋀_*y*∈*V*_{*g*_*R*_*i*__(*x*, *y*)∨(*h*_*A*_(*y*)∧*h*_*A*′_(*y*))}, ⋀_*i*=1_^*m*^⋁_*y*∈*V*_{*h*_*R*_*i*__(*x*, *y*)∧(*g*_*A*_(*y*)∧*g*_*A*′_(*y*))}〉∣*x* ∈ *U*} =  {〈*x*, ⋁_*i*=1_^*m*^⋀_*y*∈*V*_{(*g*_*R*_*i*__(*x*, *y*)∨*h*_*A*_(*y*))∧(*g*_*R*_*i*__(*x*, *y*)∧*h*_*A*′_(*y*))}, ⋀_*i*=1_^*m*^⋁_*y*∈*V*_{(*h*_*R*_*i*__(*x*, *y*)∧*g*_*A*_(*y*))∧(*h*_*R*_*i*__(*x*, *y*)∧*g*_*A*′_(*y*))}〉∣*x* ∈ *U*} =  {〈*x*, ⋁_*i*=1_^*m*^⋀_*y*∈*V*_{*g*_*R*_*i*__(*x*, *y*)∨*h*_*A*_(*y*)}, ⋀_*i*=1_^*m*^⋁_*y*∈*V*_{*h*_*R*_*i*__(*x*, *y*)∧*g*_*A*_(*y*)}〉∣*x* ∈ *U*}∧ {〈*x*, ⋁_*i*=1_^*m*^⋀_*y*∈*V*_{*g*_*R*_*i*__(*x*, *y*)∨*h*_*A*′_(*y*)}, ⋀_*i*=1_^*m*^⋁_*y*∈*V*_{*h*_*R*_*i*__(*x*, *y*)∧*g*_*A*′_(*y*)}〉∣*x* ∈ *U*} =  ${\underline{\sum_{i=1}^{m}R_{i}}}^{O}\left(A\right)\cap {\underline{\sum_{i=1}^{m}R_{i}}}^{O}\left(A^{\prime }\right)$. Similarly, it is not difficult to prove that ${\bar{\sum_{i=1}^{m}R_{i}}}^{O}\left(A\cup A^{\prime }\right)={\bar{\sum_{i=1}^{m}R_{i}}}^{O}\left(A\right)\cup {\bar{\sum_{i=1}^{m}R_{i}}}^{O}\left(A^{\prime }\right)$.

(4) From the discussions above, it is not difficult to prove that ${\underline{\sum_{i=1}^{m}R_{i}}}^{O}\left(A\cup A^{\prime }\right)⊇$ ${\underline{\sum_{i=1}^{m}R_{i}}}^{O}\left(A\right)\cup {\underline{\sum_{i=1}^{m}R_{i}}}^{O}\left(A^{\prime }\right)$ and ${\bar{\sum_{i=1}^{m}R_{i}}}^{O}\left(A\cap A^{\prime }\right)\subseteq {\bar{\sum_{i=1}^{m}R_{i}}}^{O}\left(A\right)\cap {\bar{\sum_{i=1}^{m}R_{i}}}^{O}\left(A^{\prime }\right)$.

In the above theorem, (1) shows the complement of optimistic DHF multigranulation rough set over two universes; (2) shows the monotone of optimistic DHF multigranulation rough set over two universes with respect to the variety of dual hesitant fuzzy target; (3) and (4) show the multiplication and addition of optimistic DHF multigranulation rough set over two universes.

**Theorem 17 .**

Let *U*, *V* be two nonempty and finite universes of discourse and *R*_*i*_, *R*_*i*_′ ∈ *DHFR*(*U* × *V*)  (*i* = 1,2,…, *m*) are two dual hesitant fuzzy relations over *U* × *V*. If *R*_*i*_⊆*R*_*i*_′, for any *A* ∈ *DHF*(*V*), one has the following properties:

(1) ${\underline{\sum_{i=1}^{m}R_{i}^{\prime }}}^{O}\left(A\right)\subseteq {\underline{\sum_{i=1}^{m}R_{i}}}^{O}\left(A\right)$, for all *A* ∈ *DHF*(*V*).
(2) ${\bar{\sum_{i=1}^{m}R_{i}^{\prime }}}^{O}\left(A\right)⊇{\bar{\sum_{i=1}^{m}R_{i}}}^{O}\left(A\right)$, for all *A* ∈ *DHF*(*V*).

**Proof**

Since *R*_*i*_⊆*R*_*i*_′, then by Definitions 9 and 15, we have *h*_*R*_*i*__^*σ*(*k*)^(*x*, *y*) ≤ *h*_*R*_*i*_′_^*σ*(*k*)^(*x*, *y*) and *g*_*R*_*i*__^*σ*(*k*)^(*x*, *y*) ≥ *g*_*R*_*i*_′_^*σ*(*k*)^(*x*, *y*) for any (*x*, *y*) ∈ (*U* × *V*). So it follows that {〈*x*, ⋁_*i*=1_^*m*^⋀_*y*∈*V*_{*g*_*R*_*i*__^*σ*(*k*)^(*x*, *y*)∨*h*_*A*_^*σ*(*k*)^(*y*)}, ⋀_*i*=1_^*m*^⋁_*y*∈*V*_{*h*_*R*_*i*__^*σ*(*k*)^(*x*, *y*)∧*g*_*A*_^*σ*(*k*)^(*y*)}〉∣*x* ∈ *U*}≥ {〈*x*, ⋁_*i*=1_^*m*^⋀_*y*∈*V*_{*g*_*R*_*i*_′_^*σ*(*k*)^(*x*, *y*)∨*h*_*A*_^*σ*(*k*)^(*y*)}, ⋀_*i*=1_^*m*^⋁_*y*∈*V*_{*h*_*R*_*i*_′_^*σ*(*k*)^(*x*, *y*)∧*g*_*A*_^*σ*(*k*)^(*y*)}〉∣*x* ∈ *U*}. Hence, for each *x* ∈ *U*, we have $h_{{\underline{\sum_{i=1}^{m}R_{i}^{\prime }}}^{O}\left(A\right)}\left(x\right)⪯h_{{\underline{\sum_{i=1}^{m}R_{i}^{\prime }}}^{O}\left(A\right)}\left(x\right)$ and $g_{{\underline{\sum_{i=1}^{m}R_{i}^{\prime }}}^{O}\left(A\right)}\left(x\right)⪰g_{{\underline{\sum_{i=1}^{m}R_{i}}}^{O}\left(A\right)}\left(x\right)$, which means ${\underline{\sum_{i=1}^{m}R_{i}^{\prime }}}^{O}\left(A\right)\subseteq {\underline{\sum_{i=1}^{m}R_{i}}}^{O}\left(A\right)$. Similarly, it is not difficult to prove that ${\bar{\sum_{i=1}^{m}R_{i}^{\prime }}}^{O}\left(A\right)⊇{\bar{\sum_{i=1}^{m}R_{i}}}^{O}\left(A\right)$.

Theorem 17 indicates that the lower and upper approximations in optimistic DHF multigranulation rough set over two universes are monotonic with respect to the monotonic forms of the multiple binary DHF relations.

### 3.3. Pessimistic DHF Multigranulation Rough Set over Two Universes

**Definition 18 .**

Let *U*, *V* be two nonempty and finite universes of discourse and *R*_*i*_ ∈ DHFR(*U* × *V*)  (*i* = 1,2,…, *m*) are *m* dual hesitant fuzzy relations over *U* × *V*; the pair (*U*, *V*, *R*_*i*_) is called a dual hesitant fuzzy multigranulation approximation space over two universes. For any *A* ∈ DHF(*V*), the pessimistic lower and upper approximations of *A* with respect to (*U*, *V*, *R*_*i*_) are defined as follows:

(12) ∑i=1mRi_PA=x,h∑i=1mRi_PAx,g∑i=1mRi_PAx ∣ x∈U,∑i=1mRi¯PA=x,h∑i=1mRi¯PAx,g∑i=1mRi¯PAx ∣ x∈U,

where $h_{{\underline{\sum_{i=1}^{m}R_{i}}}^{P}\left(A\right)}\left(x\right)={⋀}_{i=1}^{m}{⋀}_{y\in V}^{}\left\{g_{R_{i}}\left(x,y\right)\vee h_{A}\left(y\right)\right\}$, $g_{{\underline{\sum_{i=1}^{m}R_{i}}}^{P}\left(A\right)}\left(x\right)=$ ⋁_*i*=1_^*m*^⋁_*y*∈*V*_{*h*_*R*_*i*__(*x*, *y*)∧*g*_*A*_(*y*)}, $h_{{\bar{\sum_{i=1}^{m}R_{i}}}^{P}\left(A\right)}\left(x\right)=$ ⋁_*i*=1_^*m*^⋁_*y*∈*V*_{*h*_*R*_*i*__(*x*, *y*)∧*h*_*A*_(*y*)}, and $g_{{\bar{\sum_{i=1}^{m}R_{i}}}^{P}\left(A\right)}\left(x\right)=$ ⋀_*i*=1_^*m*^⋀_*y*∈*V*_{*g*_*R*_*i*__(*x*, *y*)∨*g*_*A*_(*y*)}.

We call the pair $\left({\underline{\sum_{i=1}^{m}R_{i}}}^{P}\left(A\right),{\bar{\sum_{i=1}^{m}R_{i}}}^{P}\left(A\right)\right)$ a pessimistic DHF multigranulation rough set over two universes of *A* with respect to (*U*, *V*, *R*_*i*_). If ${\underline{\sum_{i=1}^{m}R_{i}}}^{P}\left(A\right)={\bar{\sum_{i=1}^{m}R_{i}}}^{P}\left(A\right)$, we call *A* pessimistic-definable in (*U*, *V*, *R*_*i*_); otherwise, *A* is pessimistic-undefinable in (*U*, *V*, *R*_*i*_). It is also noted that the pessimistic DHF multigranulation rough set over two universes will reduce to a DHF rough set over two universes if *m* = 1.

**Theorem 19 .**

Let *U*, *V* be two nonempty and finite universes of discourse and *R*_*i*_ ∈ *DHFR*(*U* × *V*)  (*i* = 1,2,…, *m*) are m dual hesitant fuzzy relations over *U* × *V*. For any *A*, *A*′ ∈ *DHF*(*V*), the pessimistic DHF multigranulation rough set over two universes has the following properties:

(1) ${\underline{\sum_{i=1}^{m}R_{i}}}^{P}\left(A^{c}\right)={\left({\bar{\sum_{i=1}^{m}R_{i}}}^{P}\left(A\right)\right)}^{c}$; ${\bar{\sum_{i=1}^{m}R_{i}}}^{P}\left(A^{c}\right)={\left({\underline{\sum_{i=1}^{m}R_{i}}}^{P}\left(A\right)\right)}^{c}$.
(2) $A\subseteq A^{\prime }\Rightarrow {\underline{\sum_{i=1}^{m}R_{i}}}^{P}\left(A\right)\subseteq {\underline{\sum_{i=1}^{m}R_{i}}}^{P}\left(A^{\prime }\right)$; $A\subseteq A^{\prime }\Rightarrow {\bar{\sum_{i=1}^{m}R_{i}}}^{P}\left(A\right)\subseteq {\bar{\sum_{i=1}^{m}R_{i}}}^{P}\left(A^{\prime }\right)$.
(3) ${\underline{\sum_{i=1}^{m}R_{i}}}^{P}\left(A\cap A^{\prime }\right)=$ ${\underline{\sum_{i=1}^{m}R_{i}}}^{P}\left(A\right)\cap {\underline{\sum_{i=1}^{m}R_{i}}}^{P}\left(A^{\prime }\right)$; ${\bar{\sum_{i=1}^{m}R_{i}}}^{P}\left(A\cup A^{\prime }\right)=$ ${\bar{\sum_{i=1}^{m}R_{i}}}^{P}\left(A\right)\cup {\bar{\sum_{i=1}^{m}R_{i}}}^{P}\left(A^{\prime }\right)$.
(4) ${\underline{\sum_{i=1}^{m}R_{i}}}^{P}\left(A\cup A^{\prime }\right)⊇$ ${\underline{\sum_{i=1}^{m}R_{i}}}^{P}\left(A\right)\cup {\underline{\sum_{i=1}^{m}R_{i}}}^{P}\left(A^{\prime }\right)$; ${\bar{\sum_{i=1}^{m}R_{i}}}^{P}\left(A\cap A^{\prime }\right)\subseteq {\bar{\sum_{i=1}^{m}R_{i}}}^{P}\left(A\right)\cap$ ${\bar{\sum_{i=1}^{m}R_{i}}}^{P}\left(A^{\prime }\right)$.

In the above theorem, (1) shows the complement of pessimistic DHF multigranulation rough set over two universes; (2) shows the monotone of pessimistic DHF multigranulation rough set over two universes with respect to the variety of dual hesitant fuzzy target; (3) and (4) show the multiplication and addition of pessimistic DHF multigranulation rough set over two universes.

**Theorem 20 .**

Let *U*, *V* be two nonempty and finite universes of discourse and *R*_*i*_, *R*_*i*_′ ∈ *DHFR*(*U* × *V*)  (*i* = 1,2,…, *m*) are two dual hesitant fuzzy relations over *U* × *V*. If *R*_*i*_⊆*R*_*i*_′, for any *A* ∈ *DHF*(*V*), one has the following properties:

(1) ${\underline{\sum_{i=1}^{m}R_{i}^{\prime }}}^{P}\left(A\right)\subseteq {\underline{\sum_{i=1}^{m}R_{i}}}^{P}\left(A\right)$, for all *A* ∈ *DHF*(*V*).
(2) ${\bar{\sum_{i=1}^{m}R_{i}^{\prime }}}^{P}\left(A\right)⊇{\bar{\sum_{i=1}^{m}R_{i}}}^{P}\left(A\right)$, for all *A* ∈ *DHF*(*V*).

Theorem 20 indicates that the lower and upper approximations in pessimistic DHF multigranulation rough set over two universes are monotonic with respect to the monotonic forms of the multiple binary DHF relations.

### 3.4. The Relation between Optimistic and Pessimistic DHF Multigranulation Rough Sets over Two Universes

**Theorem 21 .**

Let *U*, *V* be two nonempty and finite universes of discourse and *R*_*i*_ ∈ *DHFR*(*U* × *V*)  (*i* = 1,2,…, *m*) are m dual hesitant fuzzy relations over *U* × *V*. For any *A* ∈ *DHF*(*V*), the DHF multigranulation rough set over two universes has the following properties:

(1) ${\underline{\sum_{i=1}^{m}R_{i}}}^{P}\left(A\right)\subseteq {\underline{\sum_{i=1}^{m}R_{i}}}^{O}\left(A\right)$.
(2) ${\bar{\sum_{i=1}^{m}R_{i}}}^{P}\left(A\right)⊇{\bar{\sum_{i=1}^{m}R_{i}}}^{O}\left(A\right)$.

**Proof**

For any *x* ∈ *U*, {〈*x*, ⋁_*i*=1_^*m*^⋀_*y*∈*V*_{*g*_*R*_*i*__^*σ*(*k*)^(*x*, *y*)∨*h*_*A*_^*σ*(*k*)^(*y*)}, ⋀_*i*=1_^*m*^⋁_*y*∈*V*_{*h*_*R*_*i*__^*σ*(*k*)^(*x*, *y*)∧*g*_*A*_^*σ*(*k*)^(*y*)}〉∣*x* ∈ *U*}≥ {〈*x*, ⋀_*i*=1_^*m*^⋀_*y*∈*V*_{*g*_*R*_*i*__^*σ*(*k*)^(*x*, *y*)∨*h*_*A*_^*σ*(*k*)^(*y*)}, ⋁_*i*=1_^*m*^⋁_*y*∈*V*_{*h*_*R*_*i*__^*σ*(*k*)^(*x*, *y*)∧*g*_*A*_^*σ*(*k*)^(*y*)}〉∣*x* ∈ *U*}. Hence, we have $h_{{\underline{\sum_{i=1}^{m}R_{i}}}^{P}\left(A\right)}\left(x\right)⪯h_{{\underline{\sum_{i=1}^{m}R_{i}}}^{O}\left(A\right)}\left(x\right)$ and $g_{{\underline{\sum_{i=1}^{m}R_{i}}}^{P}\left(A\right)}\left(x\right)⪰g_{{\underline{\sum_{i=1}^{m}R_{i}}}^{O}\left(A\right)}\left(x\right)$, which means ${\underline{\sum_{i=1}^{m}R_{i}}}^{P}\left(A\right)\subseteq {\underline{\sum_{i=1}^{m}R_{i}}}^{O}\left(A\right)$. Similarly, it is not difficult to prove that ${\bar{\sum_{i=1}^{m}R_{i}}}^{P}\left(A\right)⊇{\bar{\sum_{i=1}^{m}R_{i}}}^{O}\left(A\right)$.

From Theorem 21, it is noted that the pessimistic DHF multigranulation lower approximation is included into the optimistic DHF multigranulation lower approximation, while the optimistic DHF multigranulation upper approximation is included into the pessimistic DHF multigranulation upper approximation.

## 4. The Approach of Medical Diagnoses

In this section, we introduce a new approach to the decision making problem in medical diagnoses by utilizing the proposed model based on DHF multigranulation rough set over two universes. The main points of our model and decision making methods can be summarized as the following steps.

### 4.1. The Application Model

Suppose that *U* = {*x*_1_, *x*_2_,…, *x*_*j*_} is a set of diagnoses and *V* = {*y*_1_, *y*_2_,…, *y*_*k*_} is a set of symptoms. Let *R*_*i*_ ∈ DHFR(*U* × *V*)  (*i* = 1,2,…, *m*) be *m* dual hesitant fuzzy relations over *U* × *V*, which reflects the medical knowledge base with dual hesitant fuzzy elements data given by *m* experts. We also let *A* ∈ DHF(*V*) be the set of symptoms characteristic for the considered patients. Then, we obtain a dual hesitant fuzzy decision information system (*U*, *V*, *R*_*i*_, *A*) in medical diagnoses.

In the following, we present an approach to the decision making for the above-mentioned problem by using DHF multigranulation rough set over two universes. At first, according to Definitions 15 and 18, we determine the lower and upper approximations of optimistic and pessimistic DHF multigranulation rough set over two universes of *A* with respect to (*U*, *V*, *R*_*i*_), respectively. That is, we obtain the set ${\underline{\sum_{i=1}^{m}R_{i}}}^{O}\left(A\right)$, ${\bar{\sum_{i=1}^{m}R_{i}}}^{O}\left(A\right)$, ${\underline{\sum_{i=1}^{m}R_{i}}}^{P}\left(A\right)$, and ${\bar{\sum_{i=1}^{m}R_{i}}}^{P}\left(A\right)$. Then, according to the operational laws presented in [8], *d*_1_(*x*) ⊕ *d*_2_(*x*) =  ∪_*γ*_*d*_1_(*x*)_∈*h*_*d*_1_(*x*)_,*η*_*d*_1_(*x*)_∈*g*_*d*_1_(*x*)_,*γ*_*d*_2_(*x*)_∈*h*_*d*_2_(*x*)_,*η*_*d*_2_(*x*)_∈*g*_*d*_2_(*x*)__{{*γ*_*d*_1_(*x*)_ + *γ*_*d*_2_(*x*)_ − *γ*_*d*_1_(*x*)_ *γ*_*d*_2_(*x*)_}, {*η*_*d*_1_(*x*)_ *η*_*d*_2_(*x*)_}}, we further obtain the set of ${\underline{\sum_{i=1}^{m}R_{i}}}^{O}\left(A\right)⊕{\bar{\sum_{i=1}^{m}R_{i}}}^{O}\left(A\right)$ and ${\underline{\sum_{i=1}^{m}R_{i}}}^{P}\left(A\right)⊕{\bar{\sum_{i=1}^{m}R_{i}}}^{P}\left(A\right)$, respectively. In what follows, based on the decision making strategy developed in [23], we present the decision rules for medical diagnoses by using DHF multigranulation rough set over two universes. At first, we denote
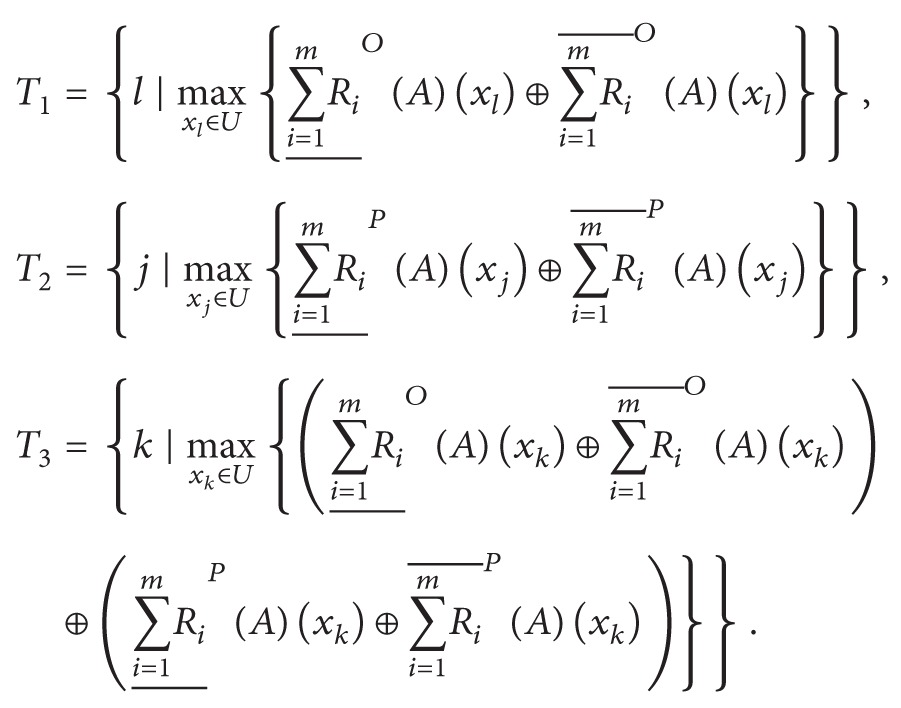
(13)


It is noted that *T*_1_, *T*_2_, and *T*_3_ indicate the decision making index sets which are composed of the subscripts of the largest dual hesitant fuzzy element in corresponding dual hesitant fuzzy sets ${\underline{\sum_{i=1}^{m}R_{i}}}^{O}\left(A\right)⊕{\bar{\sum_{i=1}^{m}R_{i}}}^{O}\left(A\right)$, ${\underline{\sum_{i=1}^{m}R_{i}}}^{P}\left(A\right)⊕{\bar{\sum_{i=1}^{m}R_{i}}}^{P}\left(A\right)$, and $\left({\underline{\sum_{i=1}^{m}R_{i}}}^{O}\left(A\right)⊕{\bar{\sum_{i=1}^{m}R_{i}}}^{O}\left(A\right)\right)⊕\left({\underline{\sum_{i=1}^{m}R_{i}}}^{P}\left(A\right)⊕{\bar{\sum_{i=1}^{m}R_{i}}}^{P}\left(A\right)\right)$, respectively. By virtue of the score function introduced in Definition 7, we can obtain the ranking orders of dual hesitant fuzzy elements in the above-mentioned corresponding dual hesitant fuzzy sets. Thus, the index sets *T*_1_, *T*_2_, and *T*_3_ could be obtained. Moreover, based on the risk decision making principle of classical operational research, we could present the practical meaning for the above three index sets according to their definitions. Since the optimistic multigranulation rough set is based on “seeking common ground while reserving differences” (SCRD) strategy, which implies that one reserves both common decisions and inconsistent decisions at the same time, thus, this opinion can be seen as a risk-seeking decision making strategy. While the pessimistic multigranulation rough set is based on “seeking common ground while eliminating differences” (SCED) strategy, this strategy indicates that one reserves common decisions while deleting inconsistent decisions. Hence, this opinion can be seen as a risk-averse decision making strategy. According to the above different decision making strategies, *x*_*i*_ (*i* ∈ *T*_1_) is the optimistic diagnostic result for the considered patient, *x*_*i*_ (*i* ∈ *T*_2_) is the pessimistic diagnostic result for the considered patient, and *x*_*i*_ (*i* ∈ *T*_3_) is the weighted diagnostic result for the considered patient, where *T*_3_ is the weighted decision making index set of *T*_1_ and *T*_2_ with the weighted value 0.5. Based on the above definition, the decision rules can be presented as follows:

(1) If *T*_1_∩*T*_2_∩*T*_3_ ≠ *∅*, then *x*_*i*_ (*i* ∈ *T*_1_∩*T*_2_∩*T*_3_) is the determined diagnosis for the patient.
(2) If *T*_1_∩*T*_2_∩*T*_3_ = *∅* and *T*_1_∩*T*_2_ ≠ *∅*, then *x*_*i*_ (*i* ∈ *T*_1_∩*T*_2_) is the determined diagnosis for the patient. Otherwise, if *T*_1_∩*T*_2_∩*T*_3_ = *∅* and *T*_1_∩*T*_2_ = *∅*, then *x*_*i*_ (*i* ∈ *T*_3_) is the determined diagnosis for the patient.

In light of the above decision rules in medical diagnoses, by virtue of the decision making index sets *T*_1_, *T*_2_, and *T*_3_ which come from optimistic and pessimistic information fusion strategies based on medical expert's risk preference, the proposed decision rules could be regarded as a multifaceted diagnostic scheme through considering multiple situations. Moreover, by utilizing the multifaceted diagnostic scheme, medical experts could obtain more reasonable and accurate diagnostic results than other approaches. Hence, the decision rules provide medical experts with a more flexible access to determine the diagnostic results for the patients.

### 4.2. Algorithm for Medical Diagnoses Using DHF Multigranulation Rough Set over Two Universes

In what follows, we present an algorithm for the medical diagnoses model based on DHF multigranulation rough set over two universes as follows.

**Algorithm 1 (the medical diagnoses based on DHF multigranulation rough set over two universes).**

*Require*. The relation between the universes *U* and *V* is provided by an expert (*U*, *V*, *R*_*i*_) and a set of symptoms characteristic for the considered patients *A*.

*Ensure*. The determined diagnosis for the patient:

(1) calculate ${\underline{\sum_{i=1}^{m}R_{i}}}^{O}\left(A\right)$, ${\bar{\sum_{i=1}^{m}R_{i}}}^{O}\left(A\right)$, ${\underline{\sum_{i=1}^{m}R_{i}}}^{P}\left(A\right)$, and ${\bar{\sum_{i=1}^{m}R_{i}}}^{P}\left(A\right)$, respectively;
(2) calculate ${\underline{\sum_{i=1}^{m}R_{i}}}^{O}\left(A\right)⊕{\bar{\sum_{i=1}^{m}R_{i}}}^{O}\left(A\right)$ and ${\underline{\sum_{i=1}^{m}R_{i}}}^{P}\left(A\right)⊕{\bar{\sum_{i=1}^{m}R_{i}}}^{P}\left(A\right)$, respectively;
(3) determine the score function values for the sets ${\underline{\sum_{i=1}^{m}R_{i}}}^{O}\left(A\right)⊕{\bar{\sum_{i=1}^{m}R_{i}}}^{O}\left(A\right)$ and ${\underline{\sum_{i=1}^{m}R_{i}}}^{P}\left(A\right)⊕{\bar{\sum_{i=1}^{m}R_{i}}}^{P}\left(A\right)$, respectively;
(4) compute *T*_1_, *T*_2_, *T*_3_, *T*_1_∩*T*_2_∩*T*_3_, and *T*_1_∩*T*_2_, and confirm the determined diagnosis for the patient.

## 5. Case Study

In this section, to illustrate the efficiency of the proposed algorithm, we use a medical diagnosis problem with DHFS information which was previously studied and modeled by Farhadinia [9]. Farhadinia proposed an approach for deriving the correlation coefficient of DHFS and further researched a medical diagnosis problem by using correlation coefficient formulas. In order to enhance the accuracy and reliability of medical diagnoses, we aim to solve the problem under the background of group decision making. In group decision making, each doctor might have their own thought about the medical knowledge base which slightly differs from other medical experts, but they should have a common goal to reach the diagnoses results for the patients by consensus and unanimity. Therefore, after a detailed discussion about several aspects of fever with some related medical experts, we obtained the required medical dataset with respect to this paper from a local provincial hospital. The medical experts not only explained the relationship about various diagnoses with a set of symptoms, but also had a conversation with some patients who are suffering from the related diseases.

Let *U* = {*x*_1_, *x*_2_, *x*_3_, *x*_4_, *x*_5_} be a set of diagnoses, where *x*_*i*_ stands for viral fever, malaria, typhoid, stomach problem, and chest problem, respectively. A patient with the given values of symptoms is denoted by *V* = {*y*_1_, *y*_2_, *y*_3_, *y*_4_, *y*_5_}, where *y*_*i*_ stands for temperature, headache, cough, stomach pain, and chest pain, respectively. The medical knowledge base with DHFS data is presented in Tables 1, 2, and 3 [9]. The symptoms characteristic for the considered patient are given. We aim to seek a diagnosis for the patient by utilizing the proposed model.

In medical diagnoses, assume that we take a sample from a patient *A* with all the symptoms, which is represented by the following dual hesitant fuzzy set information:

(14) A=y1,0.6,0.2,y2,0.3,0.5,y3,0.4,0.5,y4,0.8,0.1,y5,0.3,0.6.

Following the steps of Algorithm 1, we calculate the lower and upper approximations of optimistic and pessimistic DHF multigranulation rough sets over two universes of *A* with respect to (*U*, *V*, *R*_*i*_), respectively:

(15) ∑i=13Ri_OA=x1,0.4,0.5,0.3,0.4,x2,0.6,0.2,0.3,x3,0.3,0.5,x4,0.4,0.4,0.5,x5,0.3,0.6,∑i=13Ri¯OA=x1,0.4,0.5,0.2,x2,0.6,0.1,0.2,x3,0.3,0.4,0.5,x4,0.5,0.6,0.7,0.2,0.3,x5,0.3,0.6,∑i=13Ri_PA=x1,0.4,0.5,0.3,0.4,x2,0.4,0.5,0.6,0.3,0.4,x3,0.3,0.5,x4,0.3,0.4,0.5,x5,0.3,0.6,∑i=13Ri¯PA=x1,0.5,0.6,0.1,0.2,x2,0.7,0.8,0.1,0.2,x3,0.4,0.5,0.3,0.4,x4,0.7,0.8,0.1,0.2,x5,0.4,0.5,0.5,0.6.

Then, we further obtain ${\underline{\sum_{i=1}^{m}R_{i}}}^{O}\left(A\right)\;⊕\;{\bar{\sum_{i=1}^{m}R_{i}}}^{O}\left(A\right)$ and ${\underline{\sum_{i=1}^{m}R_{i}}}^{P}\left(A\right)⊕{\bar{\sum_{i=1}^{m}R_{i}}}^{P}\left(A\right)$ as follows:

(16) ∑i=13Ri_OA⊕∑i=13Ri¯OA=x1,0.64,0.7,0.75,0.06,0.08,x2,0.84,0.02,0.03,0.04,x3,0.51,0.58,0.25,x4,0.7,0.76,0.82,0.08,0.1,0.12,0.15,x5,0.51,0.36;∑i=13Ri_PA⊕∑i=13Ri¯PA=x1,0.7,0.75,0.76,0.8,0.03,0.04,0.06,0.08,x2,0.82,0.85,0.88,0.9,0.92,0.03,0.04,0.06,0.08,x3,0.58,0.65,0.15,0.2,x4,0.79,0.82,0.86,0.88,0.05,0.1,x5,0.58,0.65,0.3,0.36.

In what follows, according to Definition 7, we calculate the score function values of dual hesitant fuzzy elements ${\underline{\sum_{i=1}^{3}R_{i}}}^{O}\left(A\right)⊕{\bar{\sum_{i=1}^{3}R_{i}}}^{O}\left(A\right)$, ${\underline{\sum_{i=1}^{3}R_{i}}}^{P}\left(A\right)⊕{\bar{\sum_{i=1}^{3}R_{i}}}^{P}\left(A\right)$, and $\left({\underline{\sum_{i=1}^{3}R_{i}}}^{O}\left(A\right)⊕{\bar{\sum_{i=1}^{3}R_{i}}}^{O}\left(A\right)\right)⊕\left({\underline{\sum_{i=1}^{3}R_{i}}}^{P}\left(A\right)⊕{\bar{\sum_{i=1}^{3}R_{i}}}^{P}\left(A\right)\right)$, respectively. The ranking results of the above-mentioned dual hesitant fuzzy sets are the same. That is, *x*_2_ > *x*_4_ > *x*_1_ > *x*_3_ > *x*_5_. Therefore, it is not difficult to obtain *T*_1_∩*T*_2_∩*T*_3_ = {2} ≠ *∅*, which means *x*_2_ is the determined diagnosis for the patient. From the arguments of the above results, we can find that the considered patient is suffering from malaria.

In the following, in order to validate the effectiveness of the proposed model based on DHF multigranulation rough set over two universes, a comparison analysis is conducted by utilizing the most commonly used aggregation operators for dual hesitant fuzzy information. As presented in [11], we let *d*_*j*_  (*j* = 1,2,…, *n*) be a collection of DHFEs and we let *w* = (1/*n*, 1/*n*,…,1/*n*)^*T*^ be the weight vector of *d*_*j*_ with the equal weight. Then we have the following aggregation operators:

1. The dual hesitant fuzzy averaging (DHFA) operator:
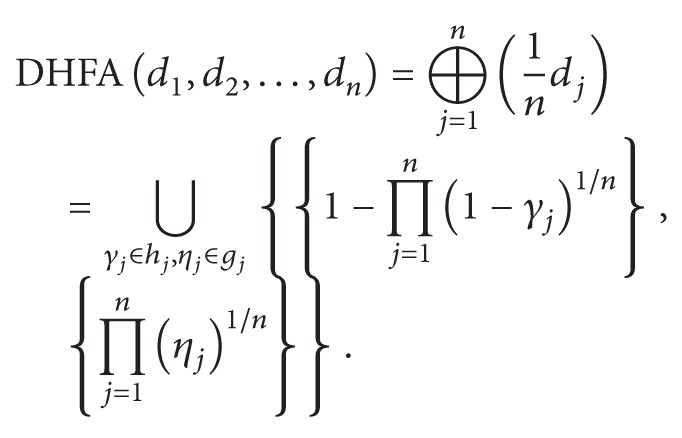
(17)
2. The dual hesitant fuzzy geometric (DHFG) operator:
  (18) DHFGd1,d2,…,dn=⨂j=1ndj1/n=⋃γj∈hj,ηj∈gj∏j=1nγj1/n,1−∏j=1n1−ηj1/n.

Through utilizing the above two aggregation operators, we can aggregate the DHF relation *R*_1_, *R*_2_, and *R*_3_ in Tables 1, 2, and 3 to a single DHF relation *R* for DHFA operator and DHFG operator, respectively. Then, within the background of dual hesitant fuzzy rough set over two universes introduced in Definition 14, we calculate the score function values of dual hesitant fuzzy elements in $\underline{R}\left(A\right)⊕\bar{R}\left(A\right)$. The ranking results for DHFA and DHFG operators are the same: *x*_2_ > *x*_4_ > *x*_1_ > *x*_3_ > *x*_5_, which is consistent with the ranking results of DHF multigranulation rough sets over two universes. Thus, the diagnostic result also shows the considered patient is suffering from malaria. In light of the above comparison analysis, though the diagnostic outcomes for the two types of information fusion strategies are indistinguishable. It is noted that the information fusion strategies for DHFA and DHFG operators are onefold. By utilizing the optimistic and pessimistic DHF multigranulation rough sets over two universes, the proposed decision rules provide a multifaceted diagnostic scheme for medical experts, which enable them to obtain more reasonable and accurate diagnostic results than DHFA and DHFG operators.

From the above analysis, the DHF multigranulation rough set over two-universe model takes full advantage of dual hesitant fuzzy set and multigranulation rough set in medical diagnoses. On one hand, compared with other generalizations of fuzzy sets, the dual hesitant fuzzy set takes into account much more information given by medical experts. That is, the nonmembership hesitancy function enables medical experts to express his or her opinions from the viewpoint of whether a patient is not suffering from a certain disease, and the hesitant information enables medical experts to hesitate among several numerical numbers when evaluating whether a patient is suffering from a certain disease or not. Thus, the dual hesitant fuzzy set provides medical experts with a more exemplary and flexible access to convey their understandings about the medical knowledge base. On the other hand, the method of multigranulation rough set is an ideal information fusion strategy which could synthesize each medical expert's view to form a final conclusion by providing optimistic and pessimistic information fusion strategies. In light of the above, the superiorities of DHF multigranulation rough set over two-universe model could decline the uncertainty to a great extent and enhance the accuracy and reliability of medical diagnoses effectively.

## 6. Conclusion

In this paper, we have proposed a new rough set model through combining multigranulation rough set and the dual hesitant fuzzy set, called a DHF multigranulation rough set over two-universe model. In this framework, the definition and some properties of optimistic and pessimistic DHF multigranulation rough sets over two universes have been studied. Finally, we have established a general approach to the decision making problem in medical diagnoses. The outcomes of the example show that the approach proposed in this paper could deal with group decision making problems effectively. Furthermore, comparing to those theoretical results in the existing literature, the main contribution of the proposed decision making model consists in taking into account three decision making index sets based on optimistic and pessimistic information fusion strategies. By virtue of the decision making index sets, the proposed decision making model provides a multifaceted diagnostic scheme for medical experts. And with the aid of multifaceted diagnostic scheme, it is convenient for medical experts to obtain more reasonable and accurate diagnostic outcomes than other methods.

This study develops a framework of DHF multigranulation rough set over two universes, in which there are still many interesting issues to be explored. In the future, we can discuss various uncertainty measures and attribute reduction approaches. It is also desirable to further apply our proposed model to other practical applications.

## Figures and Tables

**Table 1:** Symptoms characteristic for the considered diagnoses given by expert 1.

*R*_1_	*y*_1_	*y*_2_	*y*_3_	*y*_4_	*y*_5_
*x*_1_	〈{0.3,0.4}, {0.0,0.1}〉	〈{0.3,0.4}, {0.4,0.5}〉	〈{0.1,0.3}, {0.6,0.7}〉	〈{0.4,0.5}, {0.1,0.2}〉	〈{0.1,0.2}, {0.5,0.7}〉
*x*_2_	〈{0.6,0.7}, {0.0,0.1}〉	〈{0.2,0.3}, {0.4,0.6}〉	〈{0.0,0.1}, {0.8,0.9}〉	〈{0.7,0.8}, {0.0,0.2}〉	〈{0.1,0.2}, {0.7,0.8}〉
*x*_3_	〈{0.3,0.4}, {0.4,0.5}〉	〈{0.5,0.6}, {0.1,0.3}〉	〈{0.1,0.2}, {0.7,0.8}〉	〈{0.2,0.4}, {0.3,0.6}〉	〈{0.1,0.3}, {0.6,0.7}〉
*x*_4_	〈{0.1,0.3}, {0.6,0.7}〉	〈{0.2,0.3}, {0.3,0.4}〉	〈{0.6,0.8}, {0.0,0.2}〉	〈{0.6,0.7}, {0.2,0.3}〉	〈{0.2,0.3}, {0.6,0.7}〉
*x*_5_	〈{0.1,0.2}, {0.7,0.8}〉	〈{0.0,0.2}, {0.6,0.8}〉	〈{0.0,0.1}, {0.8,0.9}〉	〈{0.2,0.3}, {0.6,0.7}〉	〈{0.6,0.8}, {0.1,0.2}〉

**Table 2:** Symptoms characteristic for the considered diagnoses given by expert 2.

*R*_2_	*y*_1_	*y*_2_	*y*_3_	*y*_4_	*y*_5_
*x*_1_	〈{0.5,0.6}, {0.1,0.2}〉	〈{0.3,0.4}, {0.5,0.6}〉	〈{0.2,0.3}, {0.6,0.7}〉	〈{0.2,0.4}, {0.4,0.5}〉	〈{0.1,0.2}, {0.4,0.5}〉
*x*_2_	〈{0.6,0.7}, {0.1,0.2}〉	〈{0.3,0.4}, {0.5,0.6}〉	〈{0.2,0.3}, {0.6,0.7}〉	〈{0.5,0.6}, {0.1,0.2}〉	〈{0.3,0.4}, {0.5,0.6}〉
*x*_3_	〈{0.2,0.3}, {0.5,0.6}〉	〈{0.6,0.7}, {0.1,0.3}〉	〈{0.3,0.4}, {0.5,0.6}〉	〈{0.3,0.4}, {0.5,0.6}〉	〈{0.2,0.3}, {0.6,0.7}〉
*x*_4_	〈{0.2,0.3}, {0.5,0.6}〉	〈{0.2,0.3}, {0.4,0.5}〉	〈{0.5,0.6}, {0.1,0.2}〉	〈{0.7,0.8}, {0.1,0.2}〉	〈{0.1,0.2}, {0.6,0.7}〉
*x*_5_	〈{0.0,0.2}, {0.6,0.8}〉	〈{0.1,0.2}, {0.6,0.7}〉	〈{0.2,0.3}, {0.7,0.8}〉	〈{0.4,0.5}, {0.5,0.6}〉	〈{0.7,0.8}, {0.1,0.2}〉

**Table 3:** Symptoms characteristic for the considered diagnoses given by expert 3.

*R*_3_	*y*_1_	*y*_2_	*y*_3_	*y*_4_	*y*_5_
*x*_1_	〈{0.4,0.5}, {0.1,0.2}〉	〈{0.2,0.3}, {0.4,0.5}〉	〈{0.3,0.4}, {0.5,0.6}〉	〈{0.3,0.4}, {0.2,0.3}〉	〈{0.2,0.3}, {0.5,0.6}〉
*x*_2_	〈{0.7,0.8}, {0.1,0.2}〉	〈{0.3,0.4}, {0.5,0.6}〉	〈{0.1,0.2}, {0.7,0.8}〉	〈{0.4,0.5}, {0.0,0.2}〉	〈{0.1,0.2}, {0.7,0.8}〉
*x*_3_	〈{0.3,0.4}, {0.5,0.6}〉	〈{0.7,0.8}, {0.1,0.2}〉	〈{0.2,0.3}, {0.5,0.6}〉	〈{0.4,0.5}, {0.3,0.4}〉	〈{0.3,0.4}, {0.5,0.6}〉
*x*_4_	〈{0.3,0.4}, {0.4,0.6}〉	〈{0.1,0.3}, {0.4,0.5}〉	〈{0.4,0.5}, {0.1,0.2}〉	〈{0.5,0.7}, {0.2,0.3}〉	〈{0.2,0.3}, {0.5,0.7}〉
*x*_5_	〈{0.2,0.3}, {0.6,0.7}〉	〈{0.2,0.3}, {0.6,0.7}〉	〈{0.2,0.3}, {0.6,0.7}〉	〈{0.2,0.3}, {0.5,0.6}〉	〈{0.8,0.9}, {0.0,0.1}〉
